# Single-port versus multiport laparoscopic surgery comparing long-term patient satisfaction and cosmetic outcome

**DOI:** 10.1007/s00464-019-07351-3

**Published:** 2020-01-28

**Authors:** Jonas Raakow, Denis Klein, Atakan Görkem Barutcu, Matthias Biebl, Johann Pratschke, Roland Raakow

**Affiliations:** 1 Department of Surgery, Charité – Universitätsmedizin Berlin, Corporate Member of Freie Universität Berlin, Humboldt-Universität Zu Berlin, and Berlin Institute of Health, Charité Campus Mitte, Campus Virchow Klinikum, Charitéplatz 1, 10117 Berlin, Germany; 2 Department of General, Visceral and Vascular Surgery, Vivantes Klinikum Am Urban, Dieffenbachstrasse 1, 10967 Berlin, Germany

**Keywords:** Single-port, Laparoscopy, Cholecystectomy, Appendectomy, Cosmesis

## Abstract

**Introduction:**

Several studies and meta-analysis showed Single-port or Single-incision laparoscopic surgery (SPL) to be superior over Multiport laparoscopic surgery (MPL) mainly in terms of postoperative pain and cosmetic result. But very little is known whether these results are only a short-term effect or are persistent on the long run after SPL. We therefore evaluated and compared long-term outcomes regarding cosmesis and chronic pain after SPL and MPL.

**Methods:**

We conducted a comparative study with propensity score matching of all patients undergoing SPL or MPL between October 2008 and December 2013 in terms of postoperative cosmetic results and chronic pain. Follow-up data were obtained from mailed patient questionnaires and telephone interviews. Postoperative cosmesis was assessed using the patients overall scar opinion on a 10-point scale and the Patients scale of the standardized Patient and Observer Scar assessment scale (POSAS). Chronic pain was assessed by 10-point scales for abdominal and umbilical scar pain.

**Results:**

A total of 280 patients were included in the study with 188 patients (67.1%) after SPL and 92 patients (32.9%) following MPL. 141 patients (50.4%) underwent a cholecystectomy and 139 patients (49.6%) underwent an appendectomy. The mean follow-up time was 61.1 ± 19.1 months. The mean wound satisfaction assed by the overall scar and the PSOAS Patients scale score of the patients showed no significant difference between MPL and SPL. Patients after SPL reported more overall complains than after MPL (8.7% vs. 2.5%, respectively), but without statistical significance (*p* = 0.321). Umbilical pain scores were comparable between the two groups (1.4 ± 1.0 vs. 1.4 ± 1.0, *p* = 0.831).

**Conclusion:**

We found no difference in long-term cosmetic outcomes after SPL and MPL. Chronic pain at the umbilical incision site was comparable on the long run.

Within the last years, technical advances in minimal invasive surgery have almost made a long-standing surgical dream of widespread and technical feasible scarless operations come true. The main reason to reduce incisions and therefore scars is not only to reduce postoperative morbidity by minimalizing the incisional trauma but also to improve patient´s satisfaction with the operation mainly by improving the cosmetic outcome. But the calculation that fewer or smaller scars lead to a better cosmetic result seems too easy. More than 20 years after the initial description of the first single-incision laparoscopic cholecystectomy by Navarra et al. and the beginning of the widespread interest in this operative procedure by surgeons und the surgical industry around ten years ago it is still not accepted as a routine procedure and the results are still discussed controversially. By now, several studies and meta-analysis showed Single-port or Single-incision laparoscopic surgery (SPL) to be superior over Multiport laparoscopic surgery (MPL) mainly in terms of postoperative pain and cosmetic result [1–3]. The evidence supporting this, however, still lack real long-term data, since most studies report results of only up to 12 months postoperatively. In our opinion any new operative technique should offer advances over the highly accepted standard procedure not only within the first postoperative months but also on the long run. In the present study we therefore performed a long-term follow-up of patients that underwent SPL and MPL. The primary objective was to assess the long-term results of the cosmetic outcome, patient satisfaction with the operation and possible chronic abdominal pain or pain at the scar and to determine whether these parameters differ between patients following SPL and MPL.

## Patients and methods

All patients that underwent a single-port or a conventional multiport laparoscopic cholecystectomy or appendectomy between October 2008 and December 2013 at a single institution were contacted for a long-term follow-up by mail or telephone interview. The follow-up was approved by the Institutional review board (Ethics Committee) of the Charité – Universitätsmedizin Berlin. From August 2016, all accessible patients were first contacted by mail. Patients were contacted by telephone up to three times if no response was received. The mail and telephone questionnaire contained the same questions regarding the patients overall satisfaction with the operation and an evaluation of the cosmetic outcome. The cosmetic outcome of the scar was measured on a 10-point scale with 1 being the best result like normal skin and 10 the worst. Additionally patients were asked to fill out die Patient scale of the standardized Patient and Observer Scar assessment scale (POSAS). This Patient Scale contains six questions regarding pain, itching, color, pliability, thickness and relief of the scar. Like the overall scar evaluation the POSAS scale is a 10-point score, with 10 indicating the worst imaginable scar and 1 the best that corresponds to the situation of normal skin. The score of all six items adds up to the total score of the Patient Scale. Patients were also asked to rate pain at the umbilical scar with in the last week before the assessment using a scale scoring from 1 (no pain) to 10 (the worst pain imaginable). The POSAS scale is a very feasible and appropriate tool for the evaluation of linear scars [4–6]. Overall abdominal complains were defined as any abdominal sensation within the last week before the assessment using a scale form 1 (no complains) to 10 (the worst complains imaginable).

All patients with information regarding the follow-up were included in the study on the basis of an Intention-to-treat analysis. If patients reported about any other abdominal operation between the initial cholecystectomy or appendectomy and the date of the follow-up they were also excluded from the evaluation in the present study.

### Surgical procedures

All patients received standardized prophylactic antibiotic treatment as a single dose of cefotaxime or cefuroxime and metronidazole intravenously before the skin incision. In all multiport operations the first 10 mm trocar was placed at the umbilicus. The 10–15 mm long skin incision was made subumbilical or on the left side or directly through the umbilicus depending on the shape of the navel. Pneumoperitoneum was achieved by using the Veress needle. The multiport cholecystectomy and appendectomy were 3-port procedures with two additional 5 mm trocars. The single-port operations were mainly performed using a commercial port system (*TriPort™* or *Triport* + *™*; Olympus, Japan or *SILS™ Port*, Medtronic, USA). The single umbilical skin and fascial incisions had a length of 15–20 mm and was made at the same position as described above. In all operations the facial incisions with a length of ≥ 10 mm were closed using a slowly absorbable suture size 0. Subcutaneaus sutures were placed depending on the hight of the layer using a resorbable 3–0 suture and the skin was closed using a resorbable suture size 4–0.

### Statistical analysis

All statistical analyses were performed using SPSS (Statistical Product and Service Solutions) version 25 (IBM, New York, USA). Categorical variables were compared using crosstables and the Chi square test (*χ*^2^). Numerical continuous variables were compared by the Mann–Whitney U Test. For the comparisons of the two operative groups, the clinical features of the treatment groups (Single-port vs. Multiport laparoscopic surgery) were matched using propensity scores to decrease the potential bias caused by the non-randomized nature of this study. The covariates that were used included gender, age, the Body Mass Index (BMI), the Score of the American Society of Anesthesiologists (ASA), Comorbidities (overall) as well as Diabetes, Hypertension, and chronic obstructive pulmonary disease (COPD). The propensity scores were calculated by fitting a logistic regression model, and one-to-one matching as pairs was performed without replacement. Binary logistic regression analysis was used for calculating risk factors for an unsatisfactory cosmetic outcome with an Overall Scar opinion ≥ 5. For univariate analysis, cut points for continuous variables were set at the median of that particular variable. A *p*-value of < 0.05 was considered statistically significant.

## Results

A total of 280 patients were included in the study consisting of 206 (73.6%) female and 74 (26.4%) male patients. Mean age of all patients was 36.6 ± 16.1 years with a mean.

Body Mass Index (BMI) of 24.4 ± 4.3 kg/m^2^ and 29 patients (10.4%) being obese with a BMI ≥ 30 kg/m^2^. 188 patients (67.1%) underwent Single-Port laparoscopic (SPL) surgery and 92 patients (32.9%) Multiport laparoscopic (MPL) surgery. There was no difference between the two operative groups regarding the standard demographic parameter (Table 1). Patients in the Multiport group presented more often with the diagnosis of diabetes (14.1% vs. 3.8%; *p* = 0.004) and hypertension (25.9% vs. 11.9%; *p* = 0.007) compared to patients in the Single-port-group without a significant difference regarding overall comorbidities (27.1% vs. 33.7%; *p* = 0.266).

**Table 1:** Demographic and operative parameters

	Full cohort			Propensity score matched cohort		
	Single-port (*n* = 188)	Multiport (*n* = 92)	*p*-value	Single-port (*n* = 46)	Multiport (*n* = 46)	*p*-value
Gender						
Male	46 (24.5%)	28 (30.4%)	0.314	11 (23.9%)	11 (23.9%)	1.000
Female	142 (75.5%)	64 (69.6%)		35 (76.1%)	35 (76.1%)	
Age (years)	36.8 ± 15.4	36.2 ± 17.4	0.400	30.7 ± 13.8	29.0 ± 11.6	0.670
BMI (kg/m^2^)	24.4 ± 4.2	24.6 ± 4.5	0.709	24.1 ± 4.7	24.0 ± 4.8	0.968
Obesity (BMI ≥ 30 kg/m^2^)	19 (10.4%)	10 (11.1%)	0.838	5 (10.9%)	4 (9.1%)	1.000
ASA score (≥ III)	8 (4.3%)	4 (4.4%)	1.000	1 (2.2%)	1 (2.2%)	1.000
Comorbidities (overall)	51 (27.1%)	31 (33.7%)	0.266	6 (13.0%)	8 (17.4%)	0.773
Diabetes*	7 (3.8%)	12 (14.1%)	0.004	1 (2.2%)	1 (2.2%)	1.000
Hypertension*	22 (11.9%)	22 (25.9%)	0.004	5 (10.9%)	7 (15.2%)	0.758
COPD*	6 (3.2%)	4 (4.7%)	0.512	2 (4.3%)	0	0.495
Procedure						
Cholecystectomy	101 (53.7%)	40 (43.5%)	0.127	13 (28.3%)	15 (32.6%)	0.821
Appendectomy	87 (46.3%)	52 (56.5%)		33 (71.7%)	31 (67.4%)	
Acute Inflammation	81 (43.1%)	54 (58.7%)	0.016	30 (65.2%)	29 (63.0%)	1.000
Duration of surgery (minutes)	52.7 ± 18.3	53.8 ± 17.6	0.501	54.1 ± 21.37	52.4 ± 17.9	0.953
Additional trocar	3 (1.6%)	0	0.553	1 (2.2%)	0	1.000
Postoperative complications (overall)	9 (4.9%)	8 (8.7%)	0.285	5 (10.9%)	4 (8.7%)	1.000
SSI	2 (1.1%)	3 (3.3%)	0.337	3 (6.5%)	1 (2.2%)	0.617
Hospital stay (days)	3.4 ± 1.5	3.8 ± 1.8	0.078	3.5 ± 1.7	3.6 ± 1.5	0.412

Values as numbers and percentage or in means ± standard deviation

*BMI* Body Mass Index, *ASA* American Society of Anesthesiologists, *COPD* chronic obstructive pulmonary disease, *SSI* surgical side infection

*Multiple selection possible

141 patients (50.4%) underwent a cholecystectomy and 139 patients (49.6%) an appendectomy (Table 1). Patients presenting with signs of acute inflammation were significantly more often operated by MPL (58.7% vs. 43.1%; *p* = 0.016). The mean duration of a Cholecystectomy was 55.5 ± 18.6 min and 50.4 ± 16.7 min for an appendectomy with no significant difference between SPL and MPL. Postoperative complications were documented in 17 (6.1%) patients with 5 (1.8%) Surgical site infections (SSI) were all located at the umbilicus. There was no significant difference regarding the rate of overall morbidity and SSI between the two operative groups (Table 1). We experienced no intraoperative complications and no patient needed a reoperation. The hospital stay was shorter after SPL than after MPL (3.4 ± 1.5 days vs. 3.8 ± 1.8 days, respectively; *p* = 0.079).

The propensity score matched cohort consisted of 46 Patients in each group that underwent Single-Port or Multiport laparoscopic surgery. As shown in Table 1 there was no significant difference regarding the demographic or operative parameters between the two groups.

The mean follow-up time of all 280 patients was more than 5 years (61.1 ± 19.1 months). The mean wound satisfaction assed by the overall scar and the PSOAS Patients scale score of the patients showed no significant difference between MPL and SPL for the full as well as the propensity score matched cohort (Table 2). In the long-term umbilical pain scores were comparable between the two groups (1.4 ± 1.0 vs. 1.4 ± 1.0, p = 0.831). 96.9% of all patients would recommend the type of surgery they underwent to others and 93.7% would choose this type again.

**Table 2:** Postoperative follow-up

	Full cohort			Propensity score matched cohort		
	Single-Port (*n* = 188)	Multiport (*n* = 92)	*p*-value	Single-Port (*n* = 46)	Multiport (*n* = 46)	*p*-value
Follow-up time (months)	62.8 ± 20.8	59.5 ± 15.3	0.369	57.8 ± 20.6	56.3 ± 13.6	0.968
Overall scar opinion	1.6 ± 1.2	1.8 ± 1.7	0.628	1.7 ± 1.4	2.1 ± 2.1	0.824
POSAS patient scale	8.7 ± 5.3	8.2 ± 4.2	0.852	9.6 ± 6.2	8.6 ± 6.3	0.220
Umbilical complains	8.6%	2.5%	0.320	2.2%	7.7%	0.401
Umbilical pain	1.4 ± 1.0	1.4 ± 1.0	0.438	1.7 ± 1.6	1.5 ± 1.2	0.454
Recommend surgery to others	97.1%	96.7%	1.000	93.5%	95.1%	1.000
Choose operation again	94.7%	89.7%	0.269	95.1%	84.6%	0.242

Values as numbers and percentage or in means ± standard deviation

*POSAS* patient and observer scar assessment scale

The univariate analysis of factors influencing an unsatisfactory cosmetic outcome with an Overall Scar opinion ≥ 5 showed a postoperative SSI to be a significant parameter for the long-term cosmetic result (Table 3).

## Discussion

The central rational for the introduction of single-incision or single-port laparoscopy was the minimisation of incisions and therefore scars. This was not only driven by our surgical stimulus for technical and medical innovation or by the industry´s interest in promoting new devices and instruments but mainly by our goal to achieve higher patients satisfaction mainly by reducing postoperative pain and improving the overall cosmetic result at a comparable risk for complications. By now, several studies have shown the superiority of SPL over MPL in terms of cosmesis and postoperative pain [3, 7–10]. However, all of these studies only report a follow-up of 12 months and shorter. To the best of our knowledge, the present study is therefore the first to evaluate long-term results (over 5 years) after SPL and MPL in terms of cosmetic outcome and chronic pain.

**Table 3:** Parameters associated with unsatisfactory cosmetic outcome (Overall Scar opinion ≥ 5) in all patients (*n* = 257)

	*n*	Event	OR	95% CI	*p*-value
Sex					
Male	65	1	Reference		
Female	192	10	3.52	0.44–28.02	0.235
Age					
< 34 years	126	7	Reference		
≥ 34 years	130	4	0.54	0.15–1.89	0.355
BMI					
< 30 kg/m^2^	224	10	Reference		
≥ 30 kg/m^2^	27	1	0.82	0.10–6.69	0.855
ASA					
< III	245	11	Reference		
≥ III	11	0	–	–	–
Comorbidities*					
Overall, yes	73	1	0.24	0.03–1.89	0.174
Diabetes, yes	18	0	–	–	–
Hypertension, yes	41	0	–	–	–
COPD, yes	9	0	–	–	–
Procedure					
Cholecystectomy	132	4	Reference		
Appendectomy	125	7	1.90	0.54–6.65	0.316
Procedure					
Multiport	89	6	Reference		
Single-Port	168	5	0.42	0.13–1.43	0.167
Diagnosis					
No or Chronic Inflammation	135	5	Reference		
Acute Inflammation	122	6	1.35	0.40–4.52	0.632
Duration of surgery					
< 49 min	116	6	Reference		
≥ 49 min	140	5	0.68	0.20–2.28	0.532
Morbidity, overall					
No	240	7	Reference		
Yes	17	4	10.24	2.66–39.49	0.001
SSI					
No	257	7	Reference		
Yes	5	4	140.00	13.81–1419.65	< 0.001

Values as numbers

*BMI* Body Mass Index, *ASA* American Society of Anesthesiologists, *CI* confidence interval, *COPD* chronic obstructive pulmonary disease, *SSI* surgical side infection

*Multiple selection possible

A recent Meta-analysis by Haueter et al. showed that patient satisfaction with the scar was significantly greater following SPL with a clinically moderate to significant improvement in cosmetic scores and body image scores [2]. These results were consistent at all evaluated time intervals and were still in favor for SPL 12 months after surgery. The multicenter MUSIC trial found that patients 1 year after SPL were more pleased with their esthetic result than after MPL. Additionally they performed a standardized cosmetic evaluation by independent surgeons based on photographs of the patients´ scars. In contrast to the patients opinion the surgeons found the scar shape and the skin retraction after MPL to be esthetically more acceptable. They claimed that other not investigated factors might have influenced the patient´s opinion [1]. This underlines that the combination of multiple contributing factors, potential patients´ and observer bias, and variations in patients’ expectations contributes to difficulties in assessing cosmetic results [11]. Especially for young patients undergoing elective operations for benign indications the cosmetic result might be a significant factor on the other hand for most other patients cosmesis seems to be less concerning than the relief of symptoms, the surgeon´s reputation and avoidance of surgical complications [12–14]. It is also completely unclear whether the patient´s interest in the cosmetic results might not rather be a short- to mid-term effect around the laparoscopic operation. Bencsath et al. performed a telephone interview with patients that underwent MPL (*n* = 125) and found that at a mean follow-up time of 21 months after the multiport laparoscopy less than half of all patients (47.2%) was able to recall the correct number of incisions [15]. At a mean follow-up time of 61 months in the present work we found no difference in overall scar and the PSOAS Patients scale score between SPL and MPL. These findings might emphasize that the cosmetic difference between the two laparoscopic procedures seem to vanish on the long run.

Chronic pain after abdominal operations especially after cholecystectomy is a common sensation affecting up to over 50% of all patients [16]. A study just recently proved that early visceral pain predicts chronic pain after laparoscopic cholecystectomy [17]. With this knowledge and the fact that early postoperative pain is lower after SPL, one could assume that chronic pain must also be lower following the single-incision procedure. Christoffersen et al. addressed this question in their nationwide danish cohort study with 552 patients [18]. Around 4 years following the operation, no difference was found regarding overall chronic pain and regarding pain affecting the daily work or leisure activity between SPL and MPL. These findings go along with our results. We found the overall abdominal complains to be higher after SPL but without statistical significance and the mean pain score at the incision to be the same between the two groups. Regarding the site of the pain it is know that in multiport laparoscopy the umbilical incision is experienced as the most painful [15, 19]. And asking patients after MPL which incision to eliminate, the majority would prefer to omit the umbilical one with pain being the motivating factor for the elimination [15]. Taking these facts together this might be an explanation for our findings of comparable pain scores at the incision even though others suggest that the larger umbilical incision and the wider stretching of the tissue due to extreme positions of the instruments in SPL might induce more pain at the incision than after MPL [9].

Our present analysis also carries several limitations. First, the design was comparative but non-randomized and therefore with a possible risk of selection bias. The sample sizes of the two groups (SPL and MPL) are unequally and are not defined by a statistical sample size calculation leaving a potential risk for a type 2 error. To reduce this bias a propensity score matching was performed with the disadvantage of a smaller sample size for evaluation. Moreover, we performed a telephone and mail interview with the patients but only very few were available for a clinical examination. The cosmetic score (POSAS) is therefore only a patient score and not a two sided patient and observer score. Because the majority of the patients was fairly young and healthy undergoing an intervention for a benign indication around 5 years after the operation it was almost impossible to motivate them to attend the offered clinical examination. Finally, we do not have reliable cosmetic results of our patients in the early postoperative course and can therefore not present a comparison of short- and long-term outcomes.

## Conclusion

Overall, even though SPL seems to show superior cosmetic results up to 12 months postoperatively in other studies, the long-term outcome regarding cosmesis around 5 years following the intervention is comparable between SPL and MPL. Same with overall abdominal complains and chronic pain at the incision. However, the significance of the cosmetic result is discussed very controversially and in the end it remains a very personal opinion of the patient.
